# Practical and clinical significance of the pharmacist-led antimicrobial stewardship

**DOI:** 10.1186/s40780-026-00551-9

**Published:** 2026-02-09

**Authors:** Takumi Umemura

**Affiliations:** Department of Pharmacy, Tosei General Hospital, 160, Nishi oiwakecho, Seto, 489-8642 Aichi Japan

**Keywords:** Antimicrobial stewardship, Pharmacist, Intervention

## Abstract

Antimicrobial resistance (AMR) undermines clinical care. Thus, ensuring appropriate antimicrobial therapy is crucial in global and national AMR countermeasures. This narrative review aimed to summarize pharmacist-led antimicrobial stewardship interventions in Japan and discuss their practical and clinical significance across inpatient, outpatient, and community settings. In inpatient care, antimicrobial stewardship programs (ASPs), usually embedded within antimicrobial stewardship teams and supported by national policies and reimbursement incentives, have consistently reduced broad-spectrum antibiotic use (e.g., carbapenems). ASPs improve microbiological indicators such as *Pseudomonas aeruginosa* susceptibility while maintaining patient safety. The evidence of ASP efficiency spans large tertiary hospitals that consider post-prescription review with feedback and expanded prospective audit-and-feedback. In addition, small-to-medium hospitals that adopt pharmacist-led programs have demonstrated improved key process measures (culture acquisition, de-escalation, and duration of therapy), as well as optimized clinical outcomes in selected high-risk subgroups. Given that outpatient oral antibiotics account for a substantial proportion of total consumption, ASPs are increasingly being recognized as an essential approach. Reported pharmacist-led interventions include monitoring and feedback to high-prescription departments, regimen optimization based on susceptibility data, and dental outpatient programs that shift prescriptions from third-generation cephalosporins toward guideline-recommended penicillins. At the community level, regional educational outreach with surveillance feedback and peer-comparison approaches, as well as community pharmacy-based prescription optimization, have demonstrated meaningful shifts toward narrower-spectrum agents. Pharmacist-led ASPs in Japan have largely benefitted inpatients, whereas outpatient and community stewardship remains an essential frontier requiring enhanced implementation and rigorous assessment of patient-centered outcomes.

## Background

Antimicrobial resistance (AMR) threatens the foundation of clinical care and jeopardizes the long-term viability of global public health strategies against infectious diseases. Appropriate antimicrobial therapy is essential for the prevention and treatment of infections, safeguarding patients from life-threatening conditions and enabling safe high-risk interventions, such as surgical procedures and cancer chemotherapy. However, the common and inappropriate use of antimicrobials in healthcare and agriculture has escalated the threats, causing a global hazard. With only a few new antibiotics under development, the options for future treatment are rapidly diminishing. The absence of coordinated and urgent global action could lead to a post-antibiotic era, where once-manageable infections could regain a deadly status [1]. If effective and timely action against AMR remains lacking, by 2050, AMR could cause approximately 10 million deaths annually and result in major economic losses and social instability owing to reduced workforce productivity [2]. The World Health Organization (WHO) has proposed a five-part framework to combat AMR, which is aimed at preventing these threats: “Investing in new medicines, diagnostic tools, vaccines and other interventions,” “Improving the awareness and understanding of AMR through effective communication, education, and training,” “Optimizing the use of antimicrobial medicines in human and animal health,” “Reducing the incidence of infection through effective sanitation, hygiene and infection prevention measures, including vaccines,” and “Strengthening the knowledge and evidence base through surveillance and research.” [3]. Among these, the appropriate use of antimicrobials is a critical concern for physicians, pharmacists, and other healthcare professionals in routine clinical practice; thus, supporting guidelines have been issued in Japan [4–6]. In particular, a growing body of evidence has reported the outcomes of pharmacist-led stewardship activities, highlighting stewardship as an area requiring a significant contribution of pharmacists. Japan has generated considerable practical and clinical evidence to promote the appropriate use of antimicrobial agents. Therefore, this review aims to synthesize the available evidence on pharmacist-led antimicrobial stewardship in Japan. Furthermore, we have conducted research to generate clinical evidence in anaerobic infections [7, 8], practical evidence in support activities for the antimicrobial stewardship by pharmacists [9–11], and practical evidence for the appropriate use of voriconazole [12, 13] and related topics. Based on previous reports, this review provides an overview of pharmacist-led antimicrobial stewardship interventions in Japan, with a particular focus on clarifying their clinical significance.

## Pharmacist-led antimicrobial stewardship for inpatients

In Japan, antimicrobial stewardship has been reinforced through national AMR policy and reimbursement incentives that encourage hospitals to formalize antimicrobial stewardship teams (ASTs), where pharmacists are expected to play a central role [4]. Several studies have reported pharmacist-led antimicrobial stewardship interventions for hospitalized patients. In the subsequent sections, we will discuss the clinical implications of these interventions according to hospital bed capacity.

### Large-sized hospitals

Akazawa et al. evaluated an 8‑year stepwise stewardship program in a tertiary hospital using interrupted time‑series (ITS) analyses across three phases, namely consultation, monitoring with e‑mail feedback, and post-prescription review and feedback (PPRF), which were led by infectious diseases (ID) physicians and pharmacists [14]. The day of therapy (DOT) of carbapenems per 100 patient‑days decreased across phases (median monthly 5.46 → 3.69 → 2.78), and the PPRF phase produced a sustained reduction without clear evidence of “squeezing the balloon” toward other broad agents. In Japan, differences in stewardship staffing and institutional support (including reimbursement-driven resources) may influence implementation and outcomes, underscoring the significance of presenting institutional experience. We examined whether the full-time equivalent (FTE) of an ID pharmacist influences PPRF effectiveness [10]. After the introduction of an antimicrobial stewardship reimbursement fee, the ID pharmacist FTE increased from 0.34 to 0.63, broad‑spectrum DOT decreased, and the susceptibility of *Pseudomonas aeruginosa* improved for meropenem and levofloxacin, supporting pharmacist capacity as an implementation lever. In addition, another multicenter investigation demonstrated that institutions with sufficient FTE staffing dedicated to antimicrobial stewardship activities showed a statistically significant reduction trend in the use of broad-spectrum antibiotics [15].

Ohashi et al. expanded pharmacist‑led prospective audit and feedback (PAF) from selected antibiotics to all injectable antibiotics [16]. The mortality rate was unchanged; however, Intravenous antibiotic duration shortened, de‑escalation and appropriate dosing improved, and the methicillin-resistant *Staphylococcus aureus* (MRSA) to total *S. aureus* ratio decreased. Their subsequent 8‑year evaluation demonstrated sustained improvements, including declining MRSA proportions, improved *P. aeruginosa* susceptibility (meropenem and levofloxacin), reduced therapy duration from 2017 onward, and reduced proportion of patients receiving prolonged IV therapy [17]. Okada et al. assessed pharmacist‑driven PAF for bloodstream infection using ITS analysis and found that de‑escalation increased by 44% (95% confidence interval [CI]: 30–58), and DOT decreased for carbapenems and piperacillin/tazobactam (-16 and − 15 per 100 patient‑days, respectively), without worse 30‑day mortality [18].

Focused bundles are an efficient approach. Komatsu et al. evaluated a carbapenem program with reduced broad‑spectrum use and maintained *P. aeruginosa* susceptibility [19]. Suzuki et al. used an ITS design to assess an AST PPRF strategy focusing on carbapenem de‑escalation; carbapenem DOT decreased (median, 33.2–15.3) in all departments except for the hematology Sect [20]. These reports imply that pharmacist-led ASPs in large hospitals effectively reduce the use of broad-spectrum antibiotics such as carbapenems and improve susceptibility rates for pathogens, including *P. aeruginosa*.

### Small- and medium-sized hospitals

Several studies have reported favorable outcomes for pharmacist-led ASPs even in small- and medium-sized hospitals. In a 126‑bed community hospital, Nakamura et al. emphasized the significance of close communication among post‑prescription audit pharmacists, ward pharmacists, and the ASTs [21]. Implementation increased recommendations (10.4–21.1%), improved culture ordering (56.3–73.3%), increased de‑escalation (10.2–30.8%), and reduced carbapenem use without worsening 30‑day mortality. Furthermore, Nakano et al. conducted a pragmatic quasi-experimental study of a pharmacist-led ASP in a 260-bed secondary-care hospital (without ID physicians or an in-house microbiology laboratory) using PAF plus a pre-registration system for carbapenems [22]. After implementation, carbapenem use significantly decreased (DOT, incidence rate ratio (IRR) = 0.53 [95% CI: 0.33–0.81]), along with overall spectrum breadth for injectable antimicrobials (days of antibiotic spectrum coverage, IRR 0.87 [95% CI: 0.78–0.97]). Among all patients with bacteremia, 30-day mortality demonstrated no significant differences pre- vs. post-intervention (adjusted hazard ratio = 0.92 [95% CI: 0.56–1.51]). In contrast, in severe bacteremia (Pitt bacteremia score ≥ 4), pharmacist-driven stewardship was associated with lower 30-day mortality (adjusted hazard ratio = 0.52 [95% CI: 0.28–0.99]). Furthermore, Sawada et al. evaluated a noncertified pharmacist-led ASP in a 313-bed community hospital without ID specialists using PAF in a retrospective pre-post design [23]. After ASP implementation, the median length of stay significantly decreased from 14.8 to 13.8 days (odds ratio [OR] = 0.91 [95% CI: 0.88–0.95]). In addition, process measures improved, including shorter length of therapy (median, 6 [interquartile range (IQR) 2–10] to 6 [IQR 2–9] days; OR = 0.92 [95% CI: 0.88–0.96]) and reduced empiric use of antipseudomonal/anti-MRSA agents (27.9–25.3%; OR = 0.88 [95% CI: 0.80–0.95]). Notably, the 30-day mortality (5.1% vs. 5.1%) and 30-day readmission (5.0% vs. 5.1%) exhibited no significant differences between periods. These reports indicate that pharmacist-led antimicrobial stewardship interventions in small- to medium-sized hospitals reduce the use of broad-spectrum antimicrobials and may improve patient outcomes.

## Pharmacist-led antimicrobial stewardship for outpatients

According to a report from the AMR Clinical Reference Center in Japan, hospital antibiotic consumption in 2020 (difference-in-differences [DIDs]; defined daily doses [DDD] per 1,000 inhabitants per day) was 0.675 for inpatient parenteral antimicrobials, 0.371 for inpatient oral antimicrobials, 0.018 for outpatient parenteral antimicrobials, and 2.214 for outpatient oral agents [24]. Overall antibiotic use was higher in outpatients than in inpatients, with outpatient oral antibiotics accounting for the largest proportion. Therefore, from a macro-level perspective, optimizing antibiotic use is crucial in outpatient settings. Table 1 presents the core elements of outpatient antimicrobial stewardship [25]. The core elements for promoting appropriate antimicrobials in outpatient settings are categorized into commitment, action for policy and practice, tracking and reporting, and education and expertise. In Japan, hospital ASTs primarily focus on supporting the appropriate use of antimicrobials, mainly for parenteral agents within each facility, as described above; however, the initiatives to promote the appropriate use of antimicrobials among outpatients at their institutions are also increasingly being implemented.

**Table 1:** Core elements of outpatient antibiotic stewardship [25]

Core element	Facility-level interventions	Clinician-level interventions
**Commitment**	• Identify a single leader to direct outpatient antibiotic stewardship activities	• Write and display public commitments supporting antibiotic stewardship
	• Include stewardship duties in job descriptions / evaluation criteria	
	• Communicate with all clinic staff to align patient expectations	
**Action for policy and practice**	• Provide communication skills training for clinicians	• Use evidence-based diagnostic criteria and treatment recommendations
	• Require explicit written justification in the medical record for non-recommended antibiotic prescription	• Use delayed prescription or watchful waiting when appropriate
	• Provide support for clinical decisions (clinical decision support)	
	• Use call centers, nurse hotlines, or pharmacist consultations as triage systems to prevent unnecessary visits	
**Tracking and reporting**	• Track and report prescribing for one or more high-priority conditions	• Self-evaluate antibiotic prescription practices
	• Track and report the percentage of all visits leading to an antibiotic prescription	• Participate in CME and quality improvement activities to track and improve prescribing
	• If already doing the above, track/report complications of antibiotic use and resistance trends at the health system level	
	• Assess/share performance on quality measures and reduction goals from health plans/payers	
**Education and expertise**	• Provide face-to-face educational training (academic detailing)	• Use effective communication strategies to educate patients on when antibiotics are/are not needed
	• Provide continuing education activities for clinicians	• Educate about the potential harms of antibiotic treatment
	• Ensure timely access to persons with expertise	• Provide patient education materials

### General medical practice

In a single-center outpatient stewardship project at a Japanese university hospital, Ebihara et al. showed that routine AST activities directed at outpatients, including the monthly monitoring of oral antimicrobial use, the identification of unnecessary prescriptions for acute respiratory infections and acute diarrhea, feedback to high-prescription departments via “linked doctor” meetings, and multidisciplinary education, were associated with measurable improvements in prescriptions. Over 2 years, the total oral antibiotic consumption decreased (DDDs/1,000 outpatients 339.99 → 324.21), with marked reductions in third-generation cephalosporins (18.97 → 13.14 DDDs) and fluoroquinolones (39.12 → 33.53), and the proportion of clearly unnecessary prescriptions for targeted syndromes declined over time [26]. In outpatient settings, stewardship extends beyond syndromic management to condition-specific optimization of antimicrobial regimens and duration. We evaluated a pharmacist-led antimicrobial stewardship intervention for clarithromycin-resistant *Helicobacter pylori*, where pharmacists reviewed culture and susceptibility results and recorded (electronically) regimen recommendations before first-line eradication therapy. The comparisons of the pre-intervention (2015–2018) and stewardship (2019–2020) periods showed that initial eradication success with vonoprazan-based regimens improved from 80.0% (116 out of 145) to 94.9% (37 out of 39), while the median total drug cost per successfully eradicated patient decreased from 6,624.7 to 6,392.3 yen, reflecting decreased treatment failures and cumulative antibiotic exposure [27]. In a repeated cross-sectional study from a Japanese community hospital, Tsuchimoto et al. showed that an ASP formally targeting only inpatients nonetheless altered outpatient oral prescription patterns. Between 2013 and 2019, the proportion of appropriate outpatient treatments increased from 68.4% to 83.7%, and third-generation cephalosporin prescriptions per 1,000 outpatients decreased from 21.4 to 6.3, with compensatory increases in penicillins and co-trimoxazole. These findings suggest a “spillover” effect of inpatient-focused antimicrobial stewardship on outpatient prescription; however, the use of fluoroquinolone and prophylactic also increased [28].

### Dental practice

Tagashira Y et al. implemented a pharmacist-led intervention in a dental university hospital, providing feedback with real-time review and recommendations for in-hospital prescriptions and electronic medical record-based message feedback for outpatient (external) prescriptions, along with mini-lectures for dental students [29]. Consequently, the monthly mean proportion of prescriptions for penicillins increased from 45.6 to 84.1 per 100 oral antimicrobial prescriptions (*P* < 0.001), while that of third-generation cephalosporins decreased from 43.0 to 7.3 (*P* < 0.001) from the pre-intervention to the intervention period. Endo et al. assessed a pharmacist-led outpatient ASP in a dental/oral surgery department (pre-intervention: October 2018 to September 2020; post-intervention: October 2020 to September 2022) [30]. The intervention included distributing an antibiotic reference table (bioavailability, indications, renal-dose recommendations, costs, and guidance from the Japanese Association for Infectious Diseases/Japan Society of Chemotherapy infection guideline) and informing dentists of the baseline prescription patterns. Two pharmacists provided real-time prescription support, including recommending penicillins per guidelines, proposing Pharmacokinetics/renal function-based dosing, and suggesting alternatives for allergies (e.g., switching to clarithromycin), with additional consultation at the time of antibiotic changes/prescription. After implementation, prescription shifted from third-generation cephalosporins toward penicillins (15% → 48% for penicillins; 64% → 36% for third-generation cephalosporins; *P* < 0.05), and cefditoren pivoxil antimicrobial use density (AUD) significantly decreased from 139.3 to 82.3 (*P* < 0.05), while amoxicillin AUD increased from 22.2 to 62.7.

## Community-wide antimicrobial stewardship

Community-wide antimicrobial stewardship is essential because population-level antibiotic consumption, which is largely driven by outpatient/community prescriptions, has been associated with high rates of antimicrobial resistance, indicating that reducing unnecessary use in the community may curb resistance selection [31]. In addition, inappropriate antibiotic prescription increases patient complications, including antibiotic-associated adverse events and *Clostridioides difficile* infection, highlighting that stewardship is a core intervention for patient safety [25, 31]. Therefore, the region-wide optimization of antibiotic prescription is required to mitigate resistance pressure and reduce avoidable antibiotic-related complications. According to a report from the AMR Clinical Reference Center, the total antibiotic consumption in 2020 based on DDDs per 1,000 inhabitants per day was 3.279 in hospitals, whereas it was 7.433 in clinics (including clinics with < 19 beds); most of this consumption was attributable to oral antibiotics [32]. In a randomized control trial-based meta-analysis, audit and feedback Interventions were associated with an 11% relative reduction in antibiotic prescription volume (rate ratio [RR] = 0.89; 95% CI: 0.84–0.95), 23% relative reduction in unnecessary antibiotic initiation (*n* = 16 studies, RR = 0.77; 95% CI: 0.68– 0.87), 13% relative reduction in prolonged duration of antibiotic course (*n* = 4 studies, RR = 0.87 95% CI: 0.81–0.94), and 17% relative reduction in broad-spectrum antibiotic selection (*n* = 17 studies, RR = 0.83 95% CI: 0.75–0.93 [33]. However, analyses on the specific details or frequency of the audit-and-feedback interventions are lacking. This cross-sectional study of nationwide primary care clinics in Japan showed that higher-volume clinics, solo practices, and clinics owned by older physicians were more likely to prescribe antibiotics, particularly broad-spectrum antibiotics, for nonbacterial acute respiratory infections [34]. To promote appropriate prescription through an ASP targeting clinics outside one’s institution, a randomized control trial was used to evaluate three behavioral interventions implemented alone or in combination, namely suggested alternatives presented in electronic order sets (including nonantibiotic treatment options), accountable justification prompting clinicians to enter free-text conditions for antibiotic prescription in the electronic health records, and peer comparison emails benchmarking the prescription rates of clinicians against those of “top performers.” The study reported that accountable justification (DIDs, -7.0% [95% CI: -9.1% to -2.9%]) and peer comparison (DIDs, -5.2% [95% CI: -6.9% to -1.6%]) were more effective than the other strategy [35]. Thus, rather than indicating that static or dynamic feedback is inherently superior, these findings suggest that feedback is a key driver of behavior change. In Japan, the proportion of physicians who were unaware of the AMR Action Plan or the Manual of Antimicrobial Stewardship was approximately 40% in 2018 and remained approximately 30–35% in 2020 [36, 37]. Moreover, physicians who changed their antibiotic prescription patterns tend to be highly familiar with the AMR Action Plan and the stewardship manual [36]. In contrast, when the impact of the nationwide dissemination of The Manual of Antimicrobial Stewardship, issued by Japan’s Ministry of Health, Labor and Welfare in June 2017, was evaluated, no significant changes were detected in antibiotic-use trends among outpatients with acute respiratory tract infections or gastroenteritis [38]. This suggests that merely publishing clinical guidance is insufficient and that active efforts to promote awareness and uptake are required. Providing feedback based on surveillance data and improving the understanding of clinicians regarding the AMR Action Plan and antimicrobial stewardship guidance may facilitate appropriate antimicrobial use at the community level.

### Hospital AST-led community intervention

Inoue et al. reported that the AST-led community intervention involves lectures aimed at optimizing antimicrobial use, the regular publication of surveillance data of drug-resistant strains at the Japan Red Cross Ishikawa Hospital, and the presentation of first-line treatments for community-acquired infections [39]. Consequently, the delivery of oral antimicrobials across the region in 2020 was lower than that in 2013 by 28.7%, with the delivery of cephalosporins, quinolones, and macrolides decreasing by 34.8%, 46.8%, and 56.0%, respectively. In addition, we conducted interventions aimed at changing the physicians’ prescription patterns of oral broad-spectrum antimicrobials (particularly third-generation cephalosporins, quinolones, and macrolides) and promoting the use of narrow-spectrum agents, such as penicillins; these interventions were operationalized through eight quarterly lectures held every 3 months between April 2022 and September 2024 and delivered by ID pharmacists to the managing physicians at community clinics [11]. During these lectures, they provided an overview of Japan’s National Action Plan for appropriate antibiotic use; gave guidance on selecting antimicrobials, primarily amoxicillin, for airway bronchiolitis and acute pharyngitis per the Manual of Antimicrobial Stewardship; explained the WHO AWaRe (Access, Watch, and Reserve) classification and its significance; and presented antimicrobial use surveillance data to enable peer comparison in the Seto-Asahi area. Using ITS analysis, the DOT for penicillins showed a statistically significant increasing trend from Period 1 (prepromoting community antimicrobial stewardship) to Period 2 (promoting community antimicrobial stewardship); the slope changed was from − 0.24 to 0.13 (*P* = 0.03). The DOT for the Access group increased from 2.03 (10.7%) in Period 1 to 4.05 (18.9%) in Period 2 (*P* < 0.01). At the national level, a comprehensive evaluation that considers the AMR Action Plan, the dissemination of the Manual of Antimicrobial Stewardship, and the nationwide implementation of financial incentives has shown a downward trend in population-adjusted antibiotic prescription/consumption [40].

### Community pharmacy-led intervention

Nakamura et al. conducted a single-center quasi-experimental study in a community pharmacy (February 2021 to January 2023), where in February 2022, pharmacists began to suggest switching oral third-generation cephalosporins to guideline-recommended agents for conditions where these drugs were not first-line. After the intervention, antimicrobial consumption decreased markedly (median AUD 10.0 → 2.6; DOT 19.5 → 6.2; DDD/1000 prescriptions/month 9.1 → 2.6; all *P* < 0.001).

The proportion of antimicrobial prescriptions containing oral third-generation cephalosporins decreased from 84.7% to 19.8%, with compensatory increases in first-generation cephalosporins (0.9% → 56.6%) and penicillins (0% → 4.7%). Clinical effectiveness was maintained: antibiotic changes owing to treatment failure were 0% before and after the shift, and recurrence during follow-up decreased from 14.1% to 6.7% (*P* = 0.145) [41].

## Conclusions

Pharmacist-led antimicrobial stewardship has been implemented across inpatient, outpatient, and community settings. This narrative review provides an overview of the specific interventions and their outcomes. For inpatients, many reports have demonstrated consistent benefits, including improvements in clinical outcomes. In contrast, stewardship efforts targeting outpatients and community clinics remain insufficient, necessitating further intervention. These settings represent key areas where pharmacists are expected to play a greater role, particularly through rigorous evaluation that includes patient-centered outcomes.

## Data Availability

Not applicable.

## Abbreviations

AMR: Antimicrobial resistance
ASP: Antimicrobial stewardship program
AST: Antimicrobial stewardship teams
WHO: World Health Organization
ITS: Interrupted time‑series
PPRF: Post prescription review and feedback
ID: Infectious diseases
DOT: Day of therapy
FTE: Full time equivalent
PAF: Prospective audit and feedback
MRSA: Methicillin resistant staphylococcus aureus
CI: Confidence interval
IRR: Incidence rate ratio
DIDs: Difference in-differences
DDD: Defined daily doses
AUD: Antimicrobial use density
RR: Rate ratio
